# Strengthening training and supervisory systems to improve Skilled Birth Assistance in Jharkhand, India

**DOI:** 10.1186/1753-6561-6-S5-P2

**Published:** 2012-09-28

**Authors:** Ragini Pasricha, Manju Shukla, Madhuri Narayanan

**Affiliations:** 1 IntraHealth International, New Delhi, India

## Introduction

Jharkhand has a maternal mortality ratio of 312 maternal deaths for every 100,000 deliveries, higher than the national average of 254 maternal deaths for every 100,000 deliveries. Only 47% of the deliveries in Jharkhand are attended by a skilled birth attendant, as opposed to the national average 76% of deliveries in India, which have skilled birth attendance. There is a shortage of skilled birth attendants (SBAs) in Jharkhand. In addition, many auxiliary nurse-midwives (ANMs) have limited knowledge of pregnancy- and delivery-related complications and did not consider conducting deliveries a top job responsibility. Health subcentres lack the basic infrastructure and drugs needed by ANMs for delivery care. Most ANMs received no performance feedback and had limited support from their supervisors.

## Methods

We compare the outputs measured in January 2012 to the baseline (November 2008). We describe the intervention, its outputs and the lessons learned.

We aimed at strengthening training and supervision systems in 14 districts of Jharkhand using the following approaches, with a focus on institutionalising these into the system.

1. Use of appropriate criteria to create a pool of trainers

2. Building the capacity of trainers to provide participatory training

3. Selection of training sites based on caseload and use of a checklist to improve readiness of the site

4. Health subcentre strengthening by ensuring availability of equipment and supplies to SBAs

5. Certification of SBAs based on a minimum 80% post-test score and hands-on clinical practice

6. Rotational posting of trained SBAs at higher level health facilities to ensure skill retention

7. Medical Officers oriented on supportive supervision and tracking SBA performance using a checklist

8. Non-financial reward and recognition system to motivate well-performing SBAs

## Results

As of March 2012, 1482 SBA had been trained in 14 districts of Jharkhand with technical support from the *Vistaar* project. We have found that the newly trained SBAs conduct more institutional deliveries and provide services that adhere to standard guidelines when compared to the baseline (November 2008). In Deoghar district, the average number of institutional deliveries conducted by SBAs in the past 6 months increased from an average of 8.7 to 33.0 among SBAs who provide services at subcentres. Active management of third stage of labour including all three components - administering misoprostol, controlled cord traction and uterine massage – was non-existent during the baseline. However, nearly 97.8% of SBAs now adhere to it as a standard practice. Essential newborn care services such as early initiation of breastfeeding within an hour of birth increased from 58.0% to 95.7% of all newborns and drying and wrapping the baby to prevent hypothermia increased from 52.2% to 96.7% of all newborns.

## Discussion

Increasing skilled attendance at birth is a national priority. Despite the existence of national guidelines on training, the operationalisation of the SBA training program has remained a challenge for many state governments in India. Our project’s successful experience indicates that:

1. Utilizing government staff as trainers and strengthening training systems results in better training quality

2. Regular supervisory interaction for review of performance and feedback is critical for improved performance

3. High quality training and post training support with systematic tools are key for training to result in improved worker performance

4. Important to ensure state-level leadership and regular review of progress and infrastructural readiness for trained SBAs to practice new skills and gain confidence

## Competing interests

Authors declare that they have no conflict of interest.

## Funding statement

This study was funded by the United States Agency for International Development Cooperative Agreement 386-A-00-06-00162-00.

